# N1 enhancement in synesthesia during visual and audio–visual perception in semantic cross-modal conflict situations: an ERP study

**DOI:** 10.3389/fnhum.2014.00021

**Published:** 2014-01-30

**Authors:** Christopher Sinke, Janina Neufeld, Daniel Wiswede, Hinderk M. Emrich, Stefan Bleich, Thomas F. Münte, Gregor R. Szycik

**Affiliations:** ^1^Department of Psychiatry, Social Psychiatry and Psychotherapy, Hannover Medical SchoolHanover, Germany; ^2^Department of Neurology, University Medical Center Hamburg-EppendorfHamburg, Germany; ^3^School of Psychology and Clinical Language Sciences, University of ReadingReading, UK; ^4^Department of Neurology, University of Lübeck, Lübeck, Germany

**Keywords:** synesthesia, multimodal, EEG, N1, integration

## Abstract

Synesthesia entails a special kind of sensory perception, where stimulation in one sensory modality leads to an internally generated perceptual experience of another, not stimulated sensory modality. This phenomenon can be viewed as an abnormal multisensory integration process as here the synesthetic percept is aberrantly fused with the stimulated modality. Indeed, recent synesthesia research has focused on multimodal processing even outside of the specific synesthesia-inducing context and has revealed changed multimodal integration, thus suggesting perceptual alterations at a global level. Here, we focused on audio–visual processing in synesthesia using a semantic classification task in combination with visually or auditory–visually presented animated and in animated objects in an audio–visual congruent and incongruent manner. Fourteen subjects with auditory-visual and/or grapheme-color synesthesia and 14 control subjects participated in the experiment. During presentation of the stimuli, event-related potentials were recorded from 32 electrodes. The analysis of reaction times and error rates revealed no group differences with best performance for audio-visually congruent stimulation indicating the well-known multimodal facilitation effect. We found enhanced amplitude of the N1 component over occipital electrode sites for synesthetes compared to controls. The differences occurred irrespective of the experimental condition and therefore suggest a global influence on early sensory processing in synesthetes.

## INTRODUCTION

Synesthesia describes a specific kind of perception in which a particular stimulus in one sensory modality (“inducer”) induces a concurrent perception in another sensory modality. Each kind of synesthesia can be defined by the specific inducer-concurrent pairing. The main characteristics of synesthesia are its consistency (Baron-Cohen et al., 1987; Simner and Logie, 2007) and automaticity (Mills et al., 1999): one inducer always triggers the same concurrent sensation, which cannot be suppressed or altered voluntarily. Grapheme-color synesthesia (GCS), in which achromatic letters, words or numbers are perceived in specific colors, has been extensively investigated and is believed to be one of the most common types (Simner et al., 2006). In auditory-visual synesthesia, sounds (e.g., music or single tones) can induce additional visual experiences, such as colors, forms, and textures (Ward et al., 2006; Neufeld et al., 2012a). Usually synesthetes have multiple types of synesthesia, suggesting a more global perceptual alteration underlying synesthesia rather than a specific one that only affects specific stimuli in two sensory modalities. Recent research suggests synesthesia to be an extreme form of multisensory processing within a continuous spectrum of normal perceptual processes involving multiple senses (Bien et al., 2012). Following this point of view it is not surprising that synesthetes also show differences in multisensory processing not only restricted to the inducer-concurrent sensory modalities (Brang et al., 2012; Neufeld et al., 2012c; Sinke et al., 2012b) and that these differences are similar for both grapheme-color and audio–visual synesthetes (Neufeld et al., 2012c; Sinke et al., 2012b) indicating common sensory effects for different synesthesia phenotypes. Synesthesia is therefore not only characterized by specific synesthetic perception but rather these perceptions may be a tip of the iceberg indicating more global changes in sensory processing that are not necessary related to a specific inducer-concurrent coupling. However, up to now research on multimodal processing in synesthesia beyond typical inducer-concurrent perception is scarce.

To our knowledge, so far only three studies analyzed multimodal integration processes in synesthetes beyond the synesthetic perception (Brang et al., 2012; Neufeld et al., 2012c; Sinke et al., 2012b). Two of them focused on so-called double-flash illusion as described by Shams et al. (2000), in which a short flash is presented together with two short beep sounds while subjects have to state the number of perceived flashes. Subjects tend to report the occurrence of two flashes even though only one was presented. Regarding this effect the two mentioned studies found opposite effects: whereas Brang et al. (2012) reported an increased number of illusions in seven grapheme-color synesthetes, Neufeld et al. (2012c) found a decrease in 18 synesthetic subjects with GCS and/or auditory-visual synesthesia. The third study dedicated to this issue focused on two different multimodal effects (Sinke et al., 2012b). First, a reduced susceptibility to the so-called McGurk illusion (McGurk and MacDonald, 1976) was described in 19 synesthetes. In this illusion subjects watch a video and have to report what the person in the video says. Here the presentation of differing visual and acoustical information (video of a speaker saying “BA” dubbed with the audio track of the speaker saying “GA”) leads to the fused perception of something new (usually “DA”). In the second part of the study, audio–visual speech enhancement was found to be reduced in the synesthesia group. Previously it was shown that in a noisy environment typical subject tends to additionally rely on the visual information during speech perception (i.e., mouth movement), depending on the quality of the acoustical signal (Ross et al., 2007). Here synesthesia subjects benefit less than control subjects from viewing articulatory lip movements in acoustically compromised situations. Therefore the study of Sinke et al. (2012b) shows for the first time, that subjects affected by synesthesia have deficits related to multimodal sensory processing that are important in our everyday life, namely in the speech perception. Thus these behavioral studies suggest global differences in multimodal sensory processing in synesthesia and further – resulting from these differences specific deficits related to basic natural sensory functions like speech perception.

Common models related to synesthesia focus primarily on typical inducer-concurrent couplings. Therefore they are based on data collected within a group of synesthesia subjects characterized by one specific inducer-concurrent coupling. Thus most data stems from the most available synesthesia group – GCS – though it is rarely reported if the investigated grapheme-color synesthetes experience also additional synesthesia types. Within these models proximal and distal causes of synesthesia have been distinguished (Ward, 2013). Differences in brain connectivity have been identified as a proximal cause. For example, the well-known cross-activation model of GCS suggests unusual direct connections between anatomically adjacent brain areas responsible for processing of inducer and concurrent (Ramachandran and Hubbard, 2001). As an alternative to this feed-forward mechanism with direct connections between unimodal sensory regions, indirect mechanisms based on feedback activity have also been discussed. According to this model – the disinhibited-feedback theory – synesthesia may be caused by disinhibited feedback from higher sensory or multimodal convergence brain sites (Grossenbacher and Lovelace, 2001). One good candidate for a synesthesia-related convergence site is the intraparietal cortex (IPC). This region receives mainly multimodal input (Bremmer et al., 2001) and shows structural differences in synesthetes (Weiss and Fink, 2009). It also shows activation differences in grapheme-color (Weiss et al., 2005; Sinke et al., 2012a) and in auditory-visual synesthetes (Neufeld et al., 2012a). Furthermore inhibitory transcranial magnetic stimulation (TMS) of the IPC disrupts the synesthetic Stroop effect (Esterman et al., 2006; Muggleton et al., 2007; Rothen et al., 2010), which is usually observed in grapheme-color synesthetes (Mattingley et al., 2001; Elias et al., 2003). Recent research shows also increased functional connectivity between this area and the primary auditory and visual cortices in audio–visual synesthetes (Neufeld et al., 2012b) and with the primary visual cortex in grapheme-color synesthetes (Sinke et al., 2012a). Thus it can be expected that synesthesia and synesthesia-related deficits in multisensory integration are related to aberrations within sensory-specific and higher sensory convergence brain sites and the communication among those.

As a possible distal cause of these connectivity differences and hence synesthesia, a deficit in pruning of synaptic connections has been hypothesized (Ward, 2013). Thus, synesthesia in adults could be a result of an altered development of the whole sensory system by deficient synaptic elimination. A very recent hypothesis based on findings of the graph theoretical network analysis states that synesthetes have a generally hyper-connected brain (Hanggi et al., 2011; Jancke and Langer, 2011) which may lead to alterations in multimodal integration processes at a global level within this population (Esterman et al., 2006; Mulvenna and Walsh, 2006).

Thus recent research gives first evidence for synesthesia as a global problem of multisensory processing with perception deficits that are affecting the speech processing and which may be related to both global alterations in brain connectivity and specific changes in communication between multimodal convergence brain sites and sensory-specific areas. Very little is known about the range of the multimodal speech-related deficits in synesthesia and the underlying mechanisms. Therefore, synesthesia research focusing on speech and speech-related perception as a multimodal phenomenon is needed.

Since synesthesia subjects show performance deficits in audio–visual speech perception under noisy environment, probably related to deficits in the integration of auditory stream with matching visual information served by vocalisatory lip movements, we decided to analyze audio–visual integratory processes at the semantic level. Under the assumption that synesthesia is related to global differences in brain connectivity leading to global changes in sensory perception and resulting from development problems of the central nervous system (pruning deficit), it should be possible to find differences in performance and in brain activation at diverse processing levels during cross-modal tasks. Such differences should be independent of the type of synesthesia, as defined by the specific inducer-concurrent coupling, and should have no relation to the synesthetic perception as such. To capture these effects we decided to utilize a simple multimodal perception task using different multi- and unimodal stimuli. It is known that object detection is faster for semantically congruent multimodal stimuli compared to unimodal stimuli (multimodal facilitation effect), whereas crossmodal conflict impairs the performance (Chen and Spence, 2010). Therefore we use a categorization task including three kinds of stimuli: semantically congruent and incongruent combinations of line drawings and sounds of animated and in animated objects and visual only presentations of line drawings. To capture the brain activation related to these processes we decided to use electroencephalography (EEG). This method allows analyzing brain activity with excellent temporal resolution and therefore is predisposed to distinguish early and late effects in sensory processing in an experimental setup. For both behavioral and EEG data, we expected differences between synesthesia and control subjects with reduction of audio–visual facilitation in synesthesia within the bimodal conditions and no differences in performance pattern in the unimodal visual condition.

## MATERIALS AND METHODS

### SUBJECTS

All study subjects gave written informed consent and the study was approved by the ethics committee of the Hannover Medical School. The subjects participated voluntarily and received a small monetary recompensation for their participation.

Control subjects (*n* = 14) and synesthesia subjects (*n* = 14) were matched for age (synesthetes: 36 ± 15 years, range 19–57, controls: 36 ± 14 years, range 22–61), gender (nine women per group), and general intelligence (IQ values for synesthetes: 119 ± 13 and controls: 112 ± 17) as assessed by the MWT-B – “Mehrfach–Wortschatz Test” (Lehrl et al., 1995). Data of two synesthesia subjects had to be excluded subsequently from the analysis due to strong artifacts. All subjects were native speakers of German with normal or corrected to normal vision and reported no history of neurological or psychiatric diseases or medication.

Synesthesia was assessed during an extensive interview. After the interview, five subjects were assigned to the audio–visual synesthesia (AVS) group, six to the GCS group, and three showed both kinds of synesthesia (**Table 1** contains information regarding additional synesthetic inducer-concurrent pairings within our synesthesia population). All subjects underwent additional testing with an offline MATLAB version of the synesthesia battery (). In the battery, numbers from 0 to 9 (10) and the letters from the alphabet from A to Z (26) are presented and grapheme-color synesthetes have to select a color which matches their synesthetic experience best, while controls have to choose a color which they think fits best to the item. Additionally, we modified the battery for subjects with AVS using 36 tones similar as used by Ward et al. (2006). In this test, synesthetes are asked to choose the color which matches their experienced synesthetic color induced by the tone best, non-synesthetes are asked to select the color which they think to fit best to the tone. Subjects with both kinds of synesthesia participated in both versions of the battery. Each item of the synesthesia battery was presented three times in randomized order. To assess consistency the geometric distance in RGB color space between the three runs of each sound was calculated for each subject (Eagleman et al., 2007). More consistent color choices lead to a lower consistency score, as more consistent color choices for each sound result in more similar RGB values and thus a smaller difference between the RGB values. For grapheme-color synesthetes a threshold value of 1 was chosen as suggested by Eagleman et al. (2007). All grapheme-color synesthetes showed consistency scores lower than 1 (synesthesia group 0.59 ± 0.18, control group 2.09 ± 0.68). Since a similar threshold has not been defined for auditory-visual synesthesia, we merely show that the group of auditory-visual synesthetes was more consistent than the control group, as suggested by Ward et al. (2006). The group of audio–visual synesthetes showed significantly lower consistency scores (1.15 ± 0.45) than the control group (2.03 ± 0.47).

**Table 1:** Specification of the synesthesia subjects.

	Subjects													
Inducer-concurrent pairing	1	2	3	4	5	6^*^	7	8^*^	9	10	11	12	13	14
Grapheme-color	x	x	x	x		x	x		x	x	x			
Lexical-color	x		x	x		x	x		x	x	x			
Auditory-visual	x		x		x			x		x		x	x	x
Olfactory-visual	x	x								x				x
Gustatory-visual	x	x								x				
Pain-auditory	x									x				
Tactile-auditory	x								x					
Pain-visual		x	x										x	x
Tactile-visual										x				

*Asterisk indicates subjects excluded from the analysis.*

### STIMULI AND TASK

**FIGURE 1:**
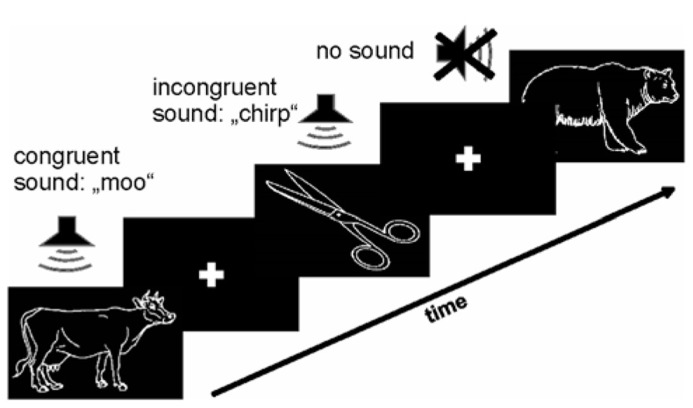
**Paradigm.** Line drawings of animate and inanimate objects (Snodgrass and Vanderwart, 1980) were combined with congruent and incongruent sounds from the MULTIMOST dataset (Schneider et al., 2008). These two conditions and an additional unimodal (visual only) control condition were presented in random order with a stimulus presentation time of 400 ms and a variable inter stimulus interval with 100 ms steps, ranging from 2 to 3 s. Subjects had to indicate animacy by pressing a button.

For visual stimulation we used line drawings of animals and inanimate objects selected from the Snodgrass and Vanderwart (1980) database presented centrally on a black screen (approximately 9° visual angle horizontally and 6° vertically). Complex natural sounds from the MULTIMOST stimulus set served as auditory stimuli (Schneider et al., 2008). Three conditions were used (**Figure 1**): in the audio–visual congruent condition the line drawing matched the presented sound (e.g., drawing of lion accompanied by a lion’s roar). This condition should lead to cross-modal enhancement. In the audio–visual incongruent condition a semantic mismatch between both modalities (e.g., a drawing of a lion accompanied by a telephone ring) was introduced. This condition is expected to lead to cross-modal conflict between visual and auditory information. The unimodal control condition comprised only visual stimulation with line drawings without sound presentation. Each stimulus was presented for duration of 400 ms. Auditory and visual stimuli in bimodal conditions were presented concurrently without time delay between onsets. The inter-stimulus interval, during which fixations cross was presented in the center of the screen, varied between 2 and 3 s in 100 ms steps. For each experimental condition (congruent, incongruent, and visual only) 80 stimuli were presented (20 different animate and 20 different inanimate stimuli, all stimuli were presented twice to increase number of events). Stimuli of all experimental categories were presented in random order. The experiment lasted about 10 min and comprised 240 stimuli in total. Participants were required to categorize each visual stimulus as either animate or inanimate as fast as possible by pressing the left or right mouse button. Before the experiment each participant completed a practice run of 10 trials with visual and acoustical congruent stimuli not presented in the main experiment to ensure that the participant understood the task. All stimuli were presented on a 19″ flat screen with a resolution of 1280 × 1024 pixels. Sounds were adjusted individually to a comfortable listening level and presented on standard loudspeakers in binaural mono. The experiment was implemented using Presentation software (Neurobehavioral Systems, Inc., Albany, CA, USA).

### DATA ACQUISITION AND PRE-PROCESSING

After application of the electrodes, participants were seated in a separate EEG recording chamber with dimmed light. Participants rested their hands on a computer mouse placed on the table in front of them, responding with their left and right index fingers. Electroencephalographic (EEG) activity was recorded continuously using an Active Two head cap and the Active Two BioSemi system (BioSemi, Amsterdam, Netherlands). Signals were recorded from 32 positions including all standard locations of the 10/20 system using active electrodes in an elastic cap. Recording of additional electrodes to record eye artifacts was not necessary, since the analysis software provides estimation of eye artifacts from frontocentral scalp electrodes (FP1, FP2). As usual for BIOSEMI, two additional electrodes (common mode sense, CMS, and driven right leg, DRL) were used as reference and ground electrodes during recording. Bioelectric signals were amplified with a sampling rate of 1024 Hz and stored using ActiView software (BioSemi) with decimation/anti-aliasing filter (5th order sinc filter, low-pass with -3 dB at 0.2035 Hz * 1024 Hz) applied to the data streamed to file. Prior to ERP analysis, EEG data were downsampled to 256 Hz and re-referenced to common average reference. We decided to use this reference method instead of re-referencing the signal to specific electrodes (e.g., averaged mastoids) because we were also interested in potential stimulation effects over the auditory cortex. A high-pass filter (1 Hz to remove low frequency drifts) and a notch filter (peak at the line frequency of 50 Hz) were applied. In the recent literature the use of high pass filtering is discussed critically. Some authors suggests to analyze data without filtering (Vanrullen, 2011) while others suggest maximal filter cut off frequency of 0.1 Hz (Acunzo et al., 2012), or values higher than 0.1 Hz (Widmann and Schroger, 2012) or lower than 1 Hz (Rousselet, 2012). Thus the use of filters affects the EEG signal in the time domain resulting in reduced precision and artifacts. We decided to use a relative high cut off frequency of 1 Hz for the high pass filter in our analysis. This relatively high value can result in some serious artifacts as shown by Acunzo et al. (2012) where the filtered signal shows artificial differences between conditions within the same experimental group. In our recent study we primary focus on differences between groups. Therefore filter settings should have the same impact on EEG signal in all experimental groups and leave the potential group difference unaffected. Indeed in studies dedicated to analysis of the impact of high pass filter setting on group differences no effects for early EEG components were found (Ebmeier et al., 1992; Goodin et al., 1992). We therefore believe that the chosen filter settings should not influence our analysis focused on group effects. Ocular contributions to the EEG were corrected using blind component separation, SOBI (Joyce et al., 2004), which has been shown to be superior to other artifact correction procedures (Kierkels et al., 2006). Rejection of non-EOG-artifacts was accomplished using individualized peak-to-peak-amplitude criteria based on visual and semi-automatic inspection implemented in BESA software (). To remove high frequency noise, ERPs were 30 Hz low-pass filtered prior to statistical analysis and graphical display. Grand-average ERPs were generated separately for both groups. ERPs were time-locked to the onset of the stimulation.

### DATA ANALYSIS

Behavior was assessed by reaction time (RT) and error rate (ER). The data was analyzed by means of 2 × 3 ANOVA with main between-subjects factor synesthesia (synesthesia vs. control group) and within-subject factor stimulation (audio–visual congruent vs. audio–visual incongruent vs. only visual stimulation).

Electroencephalography data was analyzed in two steps. The first step contained exploratory inspection of all electrodes for possible differences and relevant time windows. In the second step early ERP effects were quantified by analysis of the greatest negative peak amplitude within time window from 80 to 180 ms and late ERP effects were quantified by a mean amplitude measure between 200 and 400 ms. Since visual N1 component consist of a complex of at least three separate subcomponents that are associated with current flows over frontal (peaking at 140 ms), parietal (150 ms), and occipital (170 ms) scalp areas (Luck, 1995) analysis of this component should involve this time range of EEG signal. We decided therefore to use a time window from 80 till 180 ms used already by others for analysis (Johannes et al., 1995; Vogel and Luck, 2000) of this component. The time window for analysis of the late component was chosen to grasp possible effects on the N400 component usually modulated by semantic mismatch of the incoming information (Kutas and Federmeier, 2011). EEG data was analyzed first for global group effects and effects of stimulation with localization effects for ventro-dorsal and left-right axis. For this purpose a 2 × 3 × 2 × 4 ANOVA model was designed. This model contained one main between-subjects factor synesthesia (synesthesia vs. control group), one within-subject factor stimulation (audio–visual congruent vs. audio–visual incongruent vs. only visual stimulation) and further within-subject factors laterality (left vs. right) and electrodes (frontal vs. central vs. parietal vs. occipital) using electrodes along fronto-caudal and left-right axis (F3, C3, P3, O1, F4, C4, P4, and O2). Degrees of freedom are provided uncorrected; whenever necessary, *p*-values are Greenhouse–Geisser-corrected to account for possible violations of the sphericity assumptions.

**FIGURE 2:**
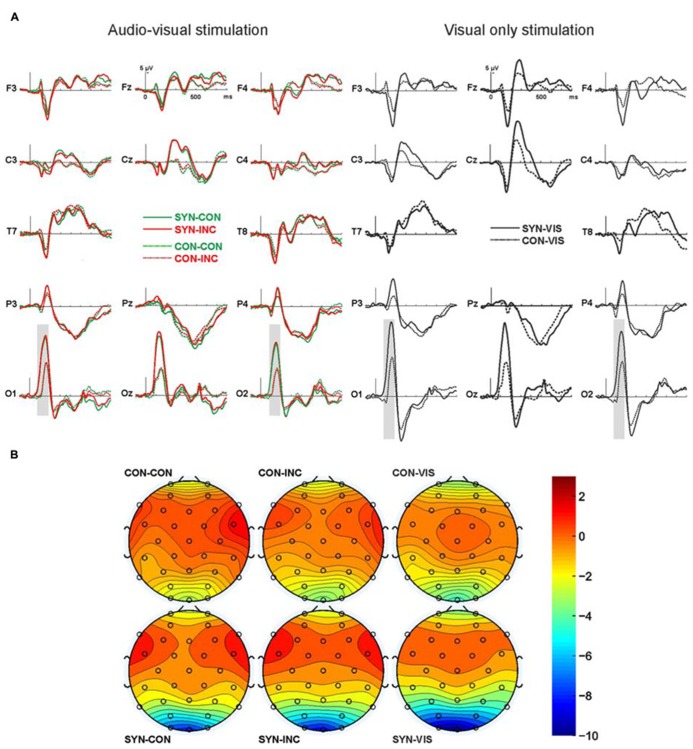
**ERPs over 14 exemplary electrodes along the left and right fronto-occipital axis. (A)** Depicted are on the left panel ERPs for audio–visual congruent (green, CON) and incongruent (red, INC) stimulation for synesthesia (continuous line, SYN) and control (dotted line, CON) subjects. On the right panel are ERPs for the unimodal visual only stimulation for synesthesia (continuous line) and control (dotted line) subjects. Electrodes are labeled according to the 10/20 system. Gray bars indicate significant differences. **(B)** Grand-mean isopotential field map for the time window from 80 to 180 ms, covering the N1 component.

Since both audio–visual conditions simultaneously included visual/auditory stimulation, both of them should show a mixed ERP based on visual and auditory potentials. In contrast, the visual stimulation control condition included visual potentials only, which enables us to see group differences when confronted with visual stimulus material only. Therefore, the visual condition is presented independently of the audio–visual conditions in the **Figure 2**.

## RESULTS

### BEHAVIORAL RESULTS

Behavioral results are summarized in **Table 2**. The ANOVA synesthesia × stimulation on ER data revealed no significant effects (stimulation *F*_2,48_ < 1, group *F*_1,24_ = 2.2 *p* = 0.15, interaction *F*_2,__48_ < 1). The ANOVA synesthesia × stimulation on response time (RT) data revealed significant effect of stimulation (*F*_2,48_ = 3.1 *p* = 0.05). Both the group factor synesthesia (*F*_1,24_ < 1) and the interaction of synesthesia with stimulation (*F*_2,48_ < 1) was not significant. *Post hoc* tests revealed faster responses for audio–visual congruent than incongruent stimuli (*t*_25_ = 2.7 *p* = 0.01) and for visual only than audio–visual incongruent (*t*_25_ = 2.1 *p* = 0.05). The difference between visual only and audio–visual congruent stimuli was not significant (*t*_25_ = 0.4 *p* = 0.72).

**Table 2:** Behavioral results.

Stimulation			
	Congruent	Incongruent	Visual
**Error rate *M*(SD)**
Control	1.1 (1.2)	1.3 (1.5)	1.2 (1.1)
Synesthesia	2.3 (2.4)	1.8 (1.8)	1.8 (1.6)
**Reaction time *M*(SD) ms**
Control	495 (73)	503 (77)	497 (63)
Synesthesia	478 (63)	484 (65)	474 (53)

### ERP RESULTS

In the overall ANOVA comparing factors synesthesia, stimulation, laterality, and electrodes for the peak analysis (N1 component) we found a significant effect of synesthesia (*F*_1,24_ = 5.4, *p* = 0.03) and a significant effect of electrodes (*F*_3,72_ = 32.4, *p* = 0.00) comparing signal from frontal (F3 and F4) vs. central (C3 and C4) vs. parietal (P3 and P4) vs. occipital (O1 and O2) electrodes. We also found a significant interaction between synesthesia and electrodes (*F*_3,72_ = 3.8, *p* = 0.04). Thus in the next step we analyzed by means of ANOVA the effects of group and stimulation in the frontal (F3, F4), central (C3, C4), parietal (P3, P4), and occipital (O1, O2) electrodes separately. We found no significant effects for the frontal, central, and parietal electrodes.

For the occipital electrodes we found a significant stimulation effect (*F*_2,48_ = 7.0, *p* = 0.08) as well as group effect (*F*_1,24_ = 5.2, *p* = 0.03) but no significant interaction between both. *Post hoc* tests revealed no significant differences in processing of audio–visual congruency (*t*-test on mean data of congruent vs. incongruent was n.s.), but congruent audio–visual vs. visual only stimulation (*t*_25_ = 2.9, *p* = 0.08, -8.4 ± 6.0 μV vs. -9.7 ± 5.8 μV) and incongruent audio–visual vs. visual only stimulation (*t*_25_ = 2.2, *p* = 0.04, -8.7 ± 5.9 μV vs. -9.7 ± 5.8 μV). Thus the described stimulation effect rises from the processing difference of multimodal vs. unimodal stimulation as such, irrespective whether multimodal stimulation was congruent or incongruent. The group effect above the occipital electrodes was due to a global difference in processing of both visual only and audio–visual stimuli with stronger negativity in the synesthesia group. We decided to calculate also *post hoc* tests for group differences although there was no significant interaction effect of both main factors. The reason for this was the relatively small synesthesia subject population analyzed. Therefore *post hoc* *t*-tests revealed a trend for significance for comparison between control and synesthesia group in audio–visual congruent condition (*t*_24_ = 1.9, *p* = 0.06, -6.3 ± 3.6 μV vs. -10.7 ± 7.5 μV) and virtually significant difference for audio-visual incongruent condition (*t*_24_ = 2.1, *p* = 0.05, -6.6 ± 3.6 vs. -11.1 ± 7.1 μV) and significant difference for visual only condition (*t*_24_ = 2.8, *p* = 0.01, -7.1 ± 3.1 vs. -12.7 ± 6.7 μV). Re-analysis using mean amplitude in a time-window from 80 to 180 ms replicated those findings.

No synesthesia (*F*_1,24_ < 1) or stimulation (*F*_2,48_ < 1) effects were found when analyzing the late components between 200 and 400 ms. The factor laterality was also not significant (*F*_1,24_ = 2.2, *p* = 0.15) but the factor electrodes showed similar to N1 component significant effect (*F*_3,72_ = 4.9, *p* = 0.03). *Post hoc* *t*-test revealed differences between the central and parietal electrodes (*t*_25_ = 5.4, *p* = 0.00, -0.1 ± 1.7 vs. 2.6 ± 2.0 μV) and between frontal and parietal electrodes (*t*_25_ = 3.7, *p* = 0.00, -0.5 ± 2.4 vs. 2.6 ± 2.0 μV).

## DISCUSSION

The aim of the study was twofold. First we were interested in global multisensory perception alterations in synesthesia independent of the specific inducer-concurrent couplings. Second we focused on neuronal activation underlying these perceptual processes. Thus, audio–visual semantic matching was used to capture multisensory processing in synesthesia at the global level. We hypothesized, based on the idea of general hyperconnectivity in synesthesia (Hanggi et al., 2011), that we would find differences in multisensory integration processes unrelated to synesthetic sensations between synesthetes and controls, i.e., in an audio–visual semantic categorization task. Particularly with regard to previous behavioral data that suggested the reduction of audio–visual integration in synesthetes as indicated by a reduced number of audio–visual illusions (Neufeld et al., 2012c; Sinke et al., 2012b) and the reduction of multisensory facilitation in speech perception (Sinke et al., 2012b), we expected global effects related to the behavior and the EEG signal.

On the behavioral level, we didn’t find any effects between groups in the experiment. Compared to controls, synesthesia subjects showed similar ERs and response speeds in audio–visual congruent and incongruent experimental events. Also, unimodal visual processing was similar in both groups. There are different possible explanations for the lack of behavioral group-effects in this study. The first one is related to the stimulation used in our experiments, i.e., it could be possible that stimulation was not able to evoke the expected effects. However, we observed in our data the well known multisensory facilitation effect (Chen and Spence, 2010) with faster responses for audio–visual congruent stimuli. Therefore our stimulation was evidently sufficient for audio–visual semantic integration. Interestingly the multisensory facilitation effect was not accompanied by significant EEG signal differences between audio–visual congruent and incongruent events. The second possibility is that previously described deficits in multisensory integration of synesthesia subjects (Neufeld et al., 2012c; Sinke et al., 2012b) are related to early processing stages involving a more basal stimulus analysis than the semantic integration/matching, which relies on the conceptual knowledge. Thus it is possible that the used stimuli in form of line drawings and complex sounds involve other integratory mechanisms than those related to temporal correspondence and spatial congruence of stimuli. Lastly, the lack of behavioral effects may be resulting from compensatory mechanisms on the neuronal level within the synesthesia group. Subjects with synesthesia might have to spend more attention to manage their interaction with the environment despite the often-reported confusion caused by synesthetic sensations. Thus they may develop strategies during their life to manage sensory input from different sensory channels separately. A good candidate for such compensatory strategy could be the control over attentional processes related to global sensory perception in synesthesia, which allows better separation of sensory information coming from different modalities. As a side effect of such strategy, or in other words, as price for successful interaction with the environment, synesthesia-specific reduction in multimodal integration could arise. Some evidence for this idea is provided by the finding that synesthesia subjects show a negative relation between their susceptibility for audio–visual illusions and their age (Neufeld et al., 2012c). This finding suggests that subjects with synesthesia are reducing their tendency to integrate multisensory information throughout their life.

Consistent with the idea of additional neuronal mechanisms related to multimodal sensory processing in synesthesia and our experimental hypothesis, we observed differences in the EEG signal of synesthetes and controls. Therefore, the main finding of this study is the global difference in the N1 negativity over occipital electrodes between synesthesia subjects and controls. This difference was unrelated to the experimental condition as synesthesia subjects showed a much stronger negativity for audio–visual congruent, incongruent as well as for unimodal visual-only stimuli. This not only indicates differences in multimodal but also in unimodal processing of synesthetic non-inducing stimuli in synesthetes and may reflect global alterations in sensory processing. Additionally we found no specific ERP effects of audio–visual congruency, neither within the synesthesia nor within the control group. Such effects were expected, since semantically congruent multimodal stimuli facilitate object detection and recognition whereas incongruent stimuli induce multimodal conflict and impair performance (Chen and Spence, 2010) and since we found this effect in the behavioral data in this study. Semantic mismatches are associated with a modulation of the so-called N400 component, which has been demonstrated for language (Kutas and Hillyard, 1980) and pictorial (Ganis and Kutas, 2003) material. The lack of audio–visual congruency/incongruency effects in our study might be explained by visual dominance effects in audio–visual conflict situations with ambiguous auditory and visual input (Yuval-Greenberg and Deouell, 2009). Another explanation for the lack of audio–visual congruency effects in the EEG signal in spite of the existence of behavioral multisensory facilitation effect in our data could be based on the small difference between the RTs for congruent vs. incongruent stimuli (about 7 ms). We consider it as possible that our experimental setting was not sufficient to capture brain correlates of such small behavioral effects. To summarize, global N1 group difference might rather be related to a generally altered visual processing in synesthetes and not to altered multisensory integration processes.

Alterations of early visual processing as measured by EEG in synesthetes have already been shown previously (Barnett et al., 2008). Barnett et al. (2008) used simple stimuli that do not elicit synesthetic color experiences and which are either mainly processed via the parvocellular or the magnocellular visual pathway. Sensory-perceptual differences in synesthetes relative to non-synesthetes in response to both types of stimuli were observed with enhanced processing of parvocellular stimuli (high contrast, high spatial frequency stimuli including color stimuli) reflected by an enhanced C1 component at 65–85 ms and a trend of decreased response to magnocellular stimuli (low contrast, low spatial frequency gray scale stimuli). The authors argue that these differences in early evoked visual potentials are a marker of widespread connectivity differences, which might be the cause of both, synesthesia as well as unrelated sensory processing differences. Alternatively, differences in early sensory processing (enhanced parvocellular and reduced magnocellular processing) might determine synesthesia by indirectly increasing a tendency to develop the paired associations of inducing stimuli with color percepts (as both, graphemes as well as colors, are more parvocellularly processed). In our study the visual stimuli were white line drawings on black background, which were therefore high in contrast and rather high in spatial frequency and therefore parvocellular in nature, which fits to the findings of Barnett et al. (2008). In line with the finding of enhanced ERPs evoked by stimuli mainly recruiting the parvocellular pathway, enhanced unimodal perception in the modality of the concurrent (which was vision or tactile sensation) has been reported in synesthetes (Banissy et al., 2009) which provides further evidence for the idea of a generally altered early unimodal processing and fits to the enhanced early N1 component over visual cortex found in our study.

Alterations of the N1 over occipitotemporal sites have been shown in grapheme-color synesthetes when exposed to numbers inducing synesthetic colors, which could either be congruent or incongruent to the meaning of a preceding sentence (Brang et al., 2008; Brang et al., 2011). More precisely, between 100 and 150 ms after onset of the sentence final stimulus (which was the number), ERPs to numbers inducing contextual congruent concurrent were more negative than ERPs to incongruent numbers in the synesthete group only. In contrast we found increased N1 component for different kinds of stimuli. Although both studies focused on different perception aspects (expectancy of content within consecutive presented sentence ending with synesthetic inducer vs. concurrent audio–visual stimulation), effects related to N1 component were found. The authors of the above mentioned study argue that their finding might indicate differences in attention shift processes dependent on the congruency of the inducers occurring on a rather early sensory level and that the observed N1 effects may reflect enhanced visual processing of contextually appropriate graphemes in the sense that fulfilled semantic expectations facilitate the grapheme discrimination as well as its synesthetic color. We go a step forward and argue that the finding of an enhanced N1 component in the current study might also be the result of an attention-related, facilitated sensory processing, but in contrast to the study by Brang et al. (2008) it occurred within the synesthesia group on a basic visual level unaffected by context (congruent or incongruent additional auditory stimuli) and without inducing synesthetic concurrents.

Influence of attention on the visual N1 in non-synesthetic individuals has already been reported in the context of both multisensory integration (Talsma and Woldorff, 2005) and unisensory visual processing (Vanvoorhis and Hillyard, 1977; Harter et al., 1982; Clark and Hillyard, 1996). Following the idea that attention already has particular impact on early stages of sensory processing in synesthesia, involvement of attention-related parietal cortex in perception could be expected. In fact, evidence for the parietal cortices key role in synesthetic perception comes from several neuroimaging studies with groups of grapheme-color synesthetes (Rouw and Scholte, 2010; Van Leeuwen et al., 2010; Sinke et al., 2012a), spatial sequence synesthetes (Tang et al., 2008) and auditory-visual synesthetes (Neufeld et al., 2012a). Importantly, connectivity analyses identified an area in the parietal cortex which showed stronger connections with primary sensory areas in synesthetes (Neufeld et al., 2012b; Sinke et al., 2012a). This is consistent with a model of parietal modulation of sensory processing which has been found to explain neuroimaging data of associator synesthetes (Van Leeuwen et al., 2011).

To summarize, in the behavioral data we found no group differences between synesthesia subjects and control subjects regarding the ER and the response speed but rather a group independent multisensory facilitation effect with faster responses for audio–visual congruent stimuli. The lack of behavioral group differences was contrasted by a global group difference in N1 for the occipital electrodes. Here, synesthesia subjects showed a stronger negativity for different kinds of stimuli. Taken together, our results give evidence for global early sensory processing alterations in synesthetes concerning a very basic level of visual processing. These early visual processing differences might either be the result of an altered connectivity within the visual cortex or of a modulation of visual processing mediated by (parietal) influences related to attention.

## Conflict of Interest Statement

The authors declare that the research was conducted in the absence of any commercial or financial relationships that could be construed as a potential conflict of interest.
